# Hospital Festivals and Meetings

**Published:** 1895-05-11

**Authors:** 


### HOSPITAL FESTIVALS AND MEETINGS.

### CITY OF LONDON HOSPITAL FOR DISEASES OF THE CHEST.

With an annual expenditure of over £11,000 a year, while the annual subscriptions only average about £2,500, and an institution whose presence in the East-end instead of the West must necessarily fail to jar the memory of the wealthy, the Managing Committee of the City of London Hospital for Diseases of the Chest might well have been pardoned for adopting a more desponding tone than that which characterised the report recently presented at the annual meeting. But a satisfactory note was sounded, the record of work done being such as would be expected from an institution which has relieved more than half-a-million of patients since the hospital was started, and that note was fully sustained at the festival dinner on Tuesday night. The Lord Chief Justice, who presided, was supported by the Hon. A. Sydney Annesley, Mr. Julian Hill (treasurer), Mr. Alderman and Sheriff Samuel, Mr. S. O. Grey, Mr. Morton Latham, Mr. C. R. Crossley, Mr. H. J. Allcroft, Dr. Thorowgood, Dr. Heron, Major Horace G. Bowen, Mr. Burdett, Mr. Vincent Harris, Mr. G. F. Stutchbury, the Rev. J. C. Mace (chaplain), Captain T. Storrar-Smith (secretary), and many representatives of the various societies which give valuable support to the hospital.

After dinner a toast list of commendable brevity was dealt with, and with such a capable speaker as Lord Russell of Killowen in the chair, it was not surprising that the post-prandial utterances attained a high level of eloquence. Sir Charles (the title comes more naturally than his later designation) departed somewhat from the beaten track when, in submitting the healths of the Prince and Princess of Wales, and the rest of the Royal Family, he referred to those who held the mistaken belief that the Prince spent his life in promoting his own enjoyment. Those who knew what the Prince's life was knew how far from the truth that supposition was.

His Lordship said he found himself in a position of which anybody might well be proud, for he knew no better position than that of being called upon to support a noble charity erected for the relief and mitigation of the sufferings of mankind — a sentiment which evoked a hearty response from the audience. Proceeding to give a short history of the hospital, Lord Russell stated the grounds upon which the appeal on behalf of the hospital was based; the Chairman went on to narrate the opinion expressed by a very "intelligent foreigner" when asked by him what it was that had struck him most in England? It was the legend (or facts behind that legend) which was inscribed on so many noble institutions in their city: "Supported by voluntary contributions." Yes; that was an intelligent foreigner! He did recognise what was to the credit of this great people, namely, that they were mindful of the cause of their less fortunate brethren. (Applause.)

At the conclusion of the Lord Chief Justice's speech, the amount of subscriptions was announced by the Secretary as £1,687 10s. The toast of "The Chairman's Health" (submitted by Mr. Morton Latham) fittingly followed the reading of the list of subscriptions, and his Lordship having replied, Mr. Burdett proposed "The President (the Duke of Connaught), the Vice-Presidents, Treasurer, and Committee." Mr. Burdett insisted that the hospital deserved support and sympathy for the reason that it was situated in a poor district, and he hoped one result of the Lord Chief Justice's presence that night in the chair would be that those who were burdened with their money, who did not know what to do with it, would go round to such charities as that hospital and see the work that was done for the suffering. If they did, they would be happier and wiser men. Mr. Burdett threw out the suggestion that a convalescent home at the Cape would be a valuable adjunct to the hospital, and concluded by coupling the name of the treasurer (Mr. Julian Hill) with the toast. That gentleman responded, and the remaining toasts were "The Corporation of the City of London," submitted by Mr. S. O. Gray, late chief accountant to the Bank of England, and acknowledged by Mr. Alderman and Sheriff Samuel; and "The Medical Staff," proposed by Mr. H. J. Allcroft, and coupled with the name of Dr. Thorowgood.

### ROYAL WESTMINSTER OPHTHALMIC HOSPITAL.

Sir Donald Stewart, G.C.B., took the chair at the festival dinner in aid of the Royal Westminster Ophthalmic Hospital, which was held on April 29th at the Hotel Metropole. A good many guests were present, amongst others the Hon. W. F. D. Smith, M.P., Major-General E. C. Sim, General Sir Donald Kimball, Colonel Sir Charles Euan Smith, and Dr. Forbes Winslow. In proposing "Prosperity to the Royal Westminster Ophthalmic Hospital," the Chairman drew attention to the fact that the institution depended entirely upon voluntary contributions, and that its structural arrangements were insufficient, a new building being, in fact, required, and he hoped that all present would put their hands in their pockets and do their utmost towards that object. Sir John Puleston gave the toast of "The Medical and Administrative Staff," and complimented the hospital on the eminence and efficiency of its members, Mr. George Cowell, F.R.C.S., and Mr. G. B. Hudson, M.P., responding. Subscriptions amounting to £778 10s. were afterwards announced.

### ROYAL HOSPITAL FOR CHILDREN AND WOMEN.

If the attendance was meagre and the oratory somewhat disappointing, the amount of the collection at the festival dinner of the Waterloo Road Hospital on Saturday, May 4th, more than supplied these deficiencies. The Hon. W. F. Danvers Smith presided over a gathering which included Sir R. H. Collins, K.C.B., Sir Frederick Wigan, Colonel the Hon. W. P. Talbot, Captain Blount, R.N., Major Roper Parkington, F.R.G.S., the Rev. A. W. Powell, M.A., LL.B., Hon. Massey Mainwaring, Dr. S. Sunderland, Dr. Gow, the Rev. G. E. Asker, Dr. Cowzens, the Rev. A. H. de Fontaine, Dr. Haig, Dr. A. S. Burham, F.R.C.S., Dr. M. Shield, M.R.C.S., &c. After the loyal and patriotic toasts, the Chairman remarking, in connection with the former, that four members of the Royal Family were patrons and patronesses of the hospital, the premier toast of the evening —"The Royal Hospital for Women and Children"—was submitted from the chair. Mr. Smith briefly summarised the history of the institution, which has treated no less than three-quarters of a million of patients since its establishment in 1816. During 1894 the number was 591 in-patients and 7,817 out-patients, with a total of 33,000 attendances. Coming to the financial aspect of the matter, Mr. Smith had the customary tale to tell of inadequate funds. At least £2,000 a year more than its ordinary income was required by the hospital, and during the past year the committee had been compelled to sell out £750 worth of Consols. From its position the hospital could not be well known, but it was the only hospital south of the Thames which could be called a special hospital for women and children. It was hoped that the building might be enlarged, but that was obviously impossible if sufficient funds were not collected to meet current expenditure. The Rev. A. W. Powell remarked that South London was far poorer than the East-end. Mr. H. M. Stanley, whose name was coupled with the toast of the officers of the institution, received a hearty welcome on rising to respond. He humorously replied to Mr. O'Connor's disparaging criticism of Waterloo Road, and said he did not know a finer street in all the "Dark Continent." Adverting to the large number of people who had been treated in the hospital since its establishment, Mr. Stanley said there were not so many in New Zealand. To think that little hospital, situated in such a sordid quarter, and by the meanest street in South London, should turn out such a magnificent total as 750,000 was perfectly miraculous. The Secretary (Mr. R. G. Kestin) responded, and read a long list of donations, including ten guineas from the Duchess of Albany, and one hundred guineas from the chairman, the total being the satisfactory amount of £1,311, and £11 11s. annual subscriptions. The toast of "The Chairman," submitted by Mr. Eastwood, concluded the list.

### KING'S COLLEGE HOSPITAL.

### Voluntaryism v. State Aid.

The opportunities Englishmen are afforded of listening to the eloquence of cultured Americans in this country are so "few and far between" that we were somewhat surprised to find empty places at the festival dinner of King's College Hospital on Wednesday in last week, when the chair was taken by the Ambassador of the United States, the Hon. Thomas M. Bayard. The gathering, however, was a very representative one, those supporting the chairman by their presence including Lord Francis Denman, the Right Hon. G. Denman, His Excellency the Japanese Minister, the Hon. W. F. D. Smith, M.P., Dean Hole, the Rev. Dr. Wace, Sir John Bridge, Dr. Lionel Beale, F.R.S., Major-General C. A. Sim, Sir William Priestley, M.D., Sir W. Paget Bowman, Bart., the Master of the Skinners' Company, the Master of the Charterhouse, Sir J. Hanham, Bart., Dr. Playfair, Professor Rose, F.R.C.S., General Moberley, Colonel Copley Wray, Dr. Burney Yeo, Sir Hugh Beevor, Bart., M.D., and the Rev. N. Bromley (warden).

In a speech which profoundly interested his auditors, as much because of its manner of delivery as for the matter it contained, Mr. Bayard made an eloquent appeal for King's College Hospital. Mr. Bayard is a tantalisingly slow speaker, but his graceful diction, and polished sentences more than repaid the patience of his listeners. He commenced by quoting Sir Michael Foster's definition of a dangerous criminal as one whose heart was wholly devoid of social duty and fatally bent on mischief, and went on to say in that assemblage he found the absolute antithesis. The feeling which originated their hospital, the feeling which continued and maintained it, was not a simple force of sentiment; it was one of the most profound and positive forces of our social safety. In his opinion it was not only the beneficence of that hospital, but the principal upon which it was based that formed its chief value to the world, and especially to the community where it was placed. It was this—that they were relying on the voluntary principle of benevolence. (Applause.) They were following not another man's view of what was right, but something more enduring—the relation and the brotherhood of mankind. From the chaplain he had received the statement that the charities and the benevolence of the institution were absolutely untrammelled by any law other than that of the brotherhood of humanity. There was no question asked of any man or woman or helpless child, but this—Do you need our aid? Nothing more democratic, nothing more humane, could be imagined than this, and how should it be supplied? Mr. Bayard went on to answer his question by pointing out that State aid would not meet the case. There must be something more, something higher than mere officialism to give that society the dignity, the importance, the true power that it possessed. (Hear, hear.) They wanted £20,000 per annum, but that was for mere machinery; it was not the motive power; the force of personal benefit, the force of individual kindness, were the forces that blended all classes of the community together and constituted the true strength of the country. It was the sense of the benefit which he bestowed which ennobled and rewarded the man who bestowed it. It was the sense of the benefits which he received which lingered in the mind of the recipient and made him a safe member of society. After stating that he had visited the hospital, Mr. Bayard said he gathered from figures furnished him by the chaplain that the 220 beds upon which suffering humanity reposed in that hospital had by the multiplication of the days of the year, and the out-patients, and by the extension of the beneficence of the laws of the hospital swollen to a mighty aggregate of more than 25,000 cases of personal relief. Why, did they know that the whole regular army of the. United States did not exceed 25,000! But the spirit that made that army small could make it large. (Hear, hear.) In conclusion, Mr. Bayard said Oliver Wendell Holmes had in graceful words drawn a picture of two armies, and the last picture he drew was of this army:

"Along its front no sabres shine,
No blood red pennons wave;
Its banner bears the single line,
'Our duty is to save.'"

As representing the army that bore upon its banner the single line, "Our duty is to save," he asked them, with full glasses and with grateful hearts, to drink "Prosperity to King's College Hospital."

Dr. Wace's name was coupled with the toast, and that gentleman made an able reply, thanking Mr. Bayard for his presence and his speech, also acknowledging the presence of the Japanese Ambassador, and quoting the opinion of an eminent surgeon that there were few things more remarkable in the war between China and Japan than the perfection of the medical and sanitary arrangements of the Japanese. Turning to the hospital and its work, Dr. Wace mentioned that 22,000 out-patients were attended to last year, besides 2,300 within its walls. In the course of an eloquent reply to the toast of "The Chairman," Mr. Bayard stated that the amount of the collection was £2,520.

### WEST HAM HOSPITAL.

The festival dinner in aid of the West Ham Hospital took place on Thursday, April 25th, at the Holborn Restaurant, Mr. E. Rider Cook presiding, and Lord Tweedmouth, Mr. Passmore Edwards, Mr. Keir Hardie, Archdeacon Stevens, and the Mayor of West Ham were present. The Chairman proposed the toast of "Prosperity to the Hospital," and pointed out that the institution, if only a small one, played an important part in a neighbourhood containing something like a quarter of a million inhabitants, the large majority of whom belonged to the working classes. The hospital had grown with the increase of the borough; starting with 39 beds in 1890, a new wing to accommodate 24 more patients had been opened the preceding week through the liberality of Mr. Passmore Edwards, who had contributed the sum of £3,000 towards its erection. The annual expenditure of the hospital was stated to be £4,000, the reliable income only amounting to £3,200, most of which was raised by special donations. Mr. Passmore Edwards duly responded, and other toasts followed. Subscriptions and donations to the amount of £713 were announced in the course of the evening.

### THE GERMAN HOSPITAL.

The jubilee festival dinner of the German Hospital, Dalston, was held on April 26th in the Whitehall Rooms of the Hotel Metropole. The Duke of Cambridge, the president of the hospital, was in the chair. There was a large gathering of guests, including Prince Christian, the German and Austro-Hungarian Ambassadors, Count Metternich, Baron von Schröder (treasurer), and Count Herman Hatzfeldt. The Duke of Cambridge, in proposing the toast of the evening, pleaded the cause of the institution, and asked earnestly for the increased help which was needed. The German Hospital accomplished an undeniably good work; it existed mainly in the interest of poor and suffering Germans in London, but no accident case was ever denied admission to its wards, and he did not think there could be any question but that it deserved every support. During the past year the in-patients treated within its walls had numbered 1,446, and the out-patients 23,422. Of the 269 cases of accidents most of the patients had been Englishmen. The total receipts for the year had amounted to £7,892, and the expenditure to £9,284; the endowment was almost nil, and further support was much needed. During the evening donations to the amount of £6,300 were announced; these including a yearly donation from the German Emperor of £200, £50 from the Emperor of Austria, and £1,000 from Baron von Schröder.

### METROPOLITAN DRINKING FOUNTAIN AND CATTLE TROUGH ASSOCIATION.

An unobtrusive but very necessary work is accomplished by this institution, and it was gratifying to find a goodly gathering at the festival dinner on Monday evening last at the Hotel Metropole. The Duke of Cambridge was to have presided, but having to dine that day with the Queen of Holland, his place was taken by Alderman Faudel Phillips, High Sheriff, whose experience at gatherings of the kind could not fail to make him an efficient chairman. He was supported by General R. W. Lowry, C.B., Sir W. H. Flower, K.C.B., General E. A. Gore, Colonel J. R. Campbell, Rev. F. W. A. Wilkinson, Colonel Tollner, Major R. H. Munn, Mr. J. Cumming Macdona, M.P., Rev. E. W. Matthews, Dr. T. A. Appleton, Major Walter Williams, Dr. T. F. MacGeagh, Captain Bell, Major Holford Daniel, Captain Sergeant, &c. The Chairman having submitted the loyal toasts, and "stifled his own feelings" by inviting the company to smoke, proposed "The Army, Navy, and Reserve Forces," to which General Lowry responded. "Prosperity to the Association" was also proposed from the chair, Mr. Phillips making, in connection with it, a speech replete with classical allusion and poetical quotation. The history of the association was, he presumed, known to them all. It commenced thirty-seven years ago by a drinking fountain being erected by the late Mr. Gurney at his own expense. "Great events from little causes spring." That fountain was now represented by some 700 drinking fountains and 700 drinking troughs for cattle. He was old enough to remember Snow Hill, with all its terrors, and he once saw five pirates of the "Flowery Land" hung on the hill on the other side. Since then we had made great progress, and nowhere more so than in regard to our drinking fountains. He drew a vivid picture of the sufferings of London horses, and pointed out that no nobler form of sympathy could exist than the endeavour to mitigate the sufferings of those who could not help themselves. For themselves they had drinking houses everywhere, but that association provided drinking fountains for those who had neither the money nor the desire to enter public-houses. Mr. Fry, the energetic treasurer of the association, responded, and said he was like "Single-speech Hamilton" in having but one speech for those occasions. He simply came to say that they were as usual "hard up" and that they represented an admirable cause. (Hear, hear, and applause.) Mr. Lewis Coward submitted the toast of "The Houses of Parliament." Defending the Lords, he pointed out how much useful work, which the public knew nothing about, was done by the members of that House on Committees; and referring to the new Speaker Mr. Coward said he had the honour of commencing his professional career in Mr. Gully's office, and he believed he would be found equal to any of those who had preceded him in the Chair. Mr. Macdona replied in a humorous speech. The Committee of Management were toasted on the proposal of Mr. J. H. Gurney. Mr. Gridley replied and alluded to the havoc done to the fountains by last winter's frost. It cost them £2,000 for extra repairs, and they had been compelled to sell out stock to meet that expenditure. The remaining toasts were, "The Chairman," proposed by Mr. J. H. Buxton; and "The Visitors," submitted by Mr. S. Waterhouse and acknowledged by Sir W. Flower. The collection realised nearly £1,000.

